# Evaluating the impact of the Oxford Brain Health Clinic pathway on psychiatrists' confidence in diagnosing memory disorders, and their perceived assessment usefulness

**DOI:** 10.1002/alz70858_100866

**Published:** 2025-12-25

**Authors:** JIAMIN DU, Chelsea Owens, Emily Giles, Urmila Keswani, Grace Gillis, Jasmine Blane, Caroline Potter, Clare E Mackay, Vanessa Raymont, Lola Martos

**Affiliations:** ^1^ University of Oxford, Oxford, Oxford, United Kingdom; ^2^ NIHR Applied Research Collaboration Oxford and Thames Valley (NIHR ARC OxTV), Oxford Health Foundation Trust, Oxford, United Kingdom; ^3^ Oxford Health NHS Foundation Trust, Oxford, United Kingdom; ^4^ Oxford Health NHS Foundation Trust, Oxford, Oxfordshire, United Kingdom; ^5^ Department of Psychiatry, Oxford Centre for Human Brain Activity (OHBA), Wellcome Centre for Integrative Neuroimaging, University of Oxford, Oxford, Oxfordshire, United Kingdom; ^6^ University of Oxford, Oxford, Oxfordshire, United Kingdom; ^7^ University of Oxford, Oxford, United Kingdom; ^8^ Wellcome Centre for Integrative Neuroimaging, University of Oxford, Oxford, United Kingdom; ^9^ Dementias Platform UK ‐ University of Oxford, Oxford, United Kingdom

## Abstract

**Background:**

The Oxford Brain Health Clinic (OBHC) was launched in August 2020 to provide enhanced assessments for memory clinic patients including MRI, advanced cognitive and lifestyle assessments, and optional research assessments. This study evaluates the impact of the OBHC pathway on psychiatrists' diagnostic confidence and the perceived usefulness of each assessment compared to a standard memory clinic (MC) pathway, to inform future refinement and NHS adoption.

**Method:**

We retrospectively selected 100 cases from the South Oxford memory clinic (50 OBHC, 50 MC) seen between August 2021 and August 2022. Anonymised records, including assessments, diagnoses, and management plans, were extracted and reviewed. Six psychiatrists rated their diagnostic confidence (0 = no confidence, 10 = no doubt) and the usefulness of each assessment using a five‐point Likert scale (range from 1 = not useful at all to 5 = extremely useful). Each case was randomly assigned to two raters.

Descriptive analysis was conducted for confidence and usefulness scores. Mann‐Whitney U tests were used to compare diagnostic confidence between pathways, and multiple linear regression was applied to adjust for the influence of patients' cognitive status.

**Results:**

In the OBHC pathway, 48% of cases had dementia, 24% had mild cognitive impairment (MCI), and 28% had no memory disorder related diagnosis, compared to 74%, 16%, and 10% respectively in the MC pathway. Mean diagnostic confidence was high in both pathways (OBHC:8.58 ± 1.2; MC:8.38 ± 1.2). Regression analysis indicated that the OBHC pathway resulted in higher diagnostic confidence (β = ‐0.521, *p* = 0.067) compared to the MC pathway, although the result was not statistically significant.

Assessments rated as useful (scores > 3) in the OBHC pathway included MRI, ACE‐III, observations, and informant interviews, while the MC pathway prioritised clinical interviews, relative discussions, CT, and MoCA.

**Conclusions:**

The OBHC pathway demonstrates diagnostic confidence equal to or greater than that of standard memory clinics and offers all patients research engagement opportunities. Some assessments within the OBHC pathway address dementia risk factors, potentially enhancing management and research initiatives despite their limited immediate diagnostic utility. These results support the further refinement of the OBHC pathway within the NHS.

